# Automated Suture Securing Technology in Mitral Valve Surgery: A Strategy to Reduce Prosthetic Dehiscence?

**DOI:** 10.1093/ejcts/ezag013

**Published:** 2026-01-09

**Authors:** Amila Kahrovic, Harald Herkner, Paul Werner, Philipp Angleitner, Iuliana Coti, Kira Osipenko, Heimo Lagler, Alfred Kocher, Marek Ehrlich, Daniel Zimpfer, Martin Andreas

**Affiliations:** Department of Cardiac and Thoracic Aortic Surgery, Medical University of Vienna, 1090 Vienna, Austria; Department of Emergency Medicine, Medical University of Vienna, 1090 Vienna, Austria; Department of Cardiac and Thoracic Aortic Surgery, Medical University of Vienna, 1090 Vienna, Austria; Department of Cardiac and Thoracic Aortic Surgery, Medical University of Vienna, 1090 Vienna, Austria; Department of Cardiac and Thoracic Aortic Surgery, Medical University of Vienna, 1090 Vienna, Austria; Department of Cardiac and Thoracic Aortic Surgery, Medical University of Vienna, 1090 Vienna, Austria; Department of Infectious Diseases and Tropical Medicine, Medical University of Vienna, 1090 Vienna, Austria; Department of Cardiac and Thoracic Aortic Surgery, Medical University of Vienna, 1090 Vienna, Austria; Department of Cardiac and Thoracic Aortic Surgery, Medical University of Vienna, 1090 Vienna, Austria; Department of Cardiac and Thoracic Aortic Surgery, Medical University of Vienna, 1090 Vienna, Austria; Department of Cardiac and Thoracic Aortic Surgery, Medical University of Vienna, 1090 Vienna, Austria; Department of Cardiac Surgery, Medical University of Graz, A-8036 Graz, Austria

**Keywords:** automated titanium fastener, hand-tied knots, prosthetic dehiscence, mitral valve surgery

## Abstract

**Objectives:**

This study aimed to assess long-term outcomes of automated titanium fasteners versus hand-tied knots in mitral valve surgery.

**Methods:**

In this retrospective, single-centre analysis, 2678 adult patients who underwent mitral valve repair or replacement between November 2008 and November 2024 at the Medical University of Vienna were included. Patients were grouped according to the suture-securing technique used: automated titanium fasteners versus hand-tied knots. The primary endpoint was prosthetic dehiscence (either mitral annuloplasty ring or valve replacement prosthesis) requiring reintervention. Secondary endpoints comprised ischaemic stroke, intracranial bleeding, and all-cause mortality during the follow-up period.

**Results:**

Among the study population, 1072 (40%) underwent mitral valve surgery using an automated titanium fastener device, and 1606 (60%) with conventional hand-tied sutures. A total of 31 patients (1.2%) had prosthetic dehiscence during the follow-up period. The risk of prosthetic dehiscence was significantly lower in the automated titanium fastener group in both univariable (crude sub-hazard ratio [sHR] 0.32; 95% confidence interval [CI], 0.12-0.86, *P* = .023) and multivariable competing risk regression analysis (adjusted sHR 0.34; 95% CI, 0.12-0.91, *P* = .033). Automated titanium fastener group was not associated with an increased risk of ischaemic stroke (adjusted sHR 0.92; 95% CI, 0.67-1.27, *P* = .600), intracranial bleeding (adjusted sHR 0.89; 95% CI, 0.52-1.52, *P* = .675), or all-cause mortality (adjusted hazard ratio 0.93; 95% CI, 0.74-1.18, *P* = .559).

**Conclusions:**

The use of an automated titanium fastener device seems to be associated with a lower risk of prosthetic dehiscence in mitral valve surgery. Due to the limited number of prosthetic dehiscence events and the potential for residual confounding, the results should be interpreted with caution.

## Graphical abstract

**Figure:**
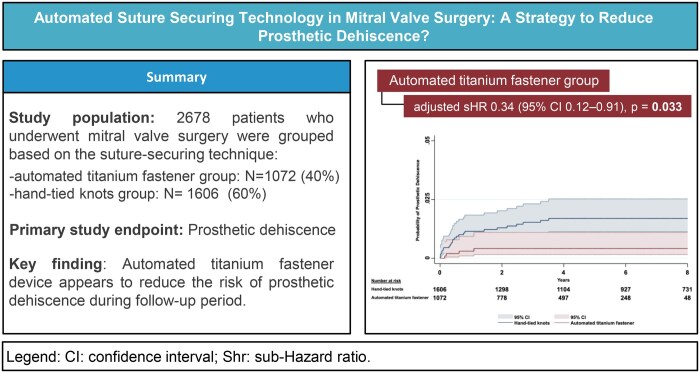

## Introduction

**Figure 1.**
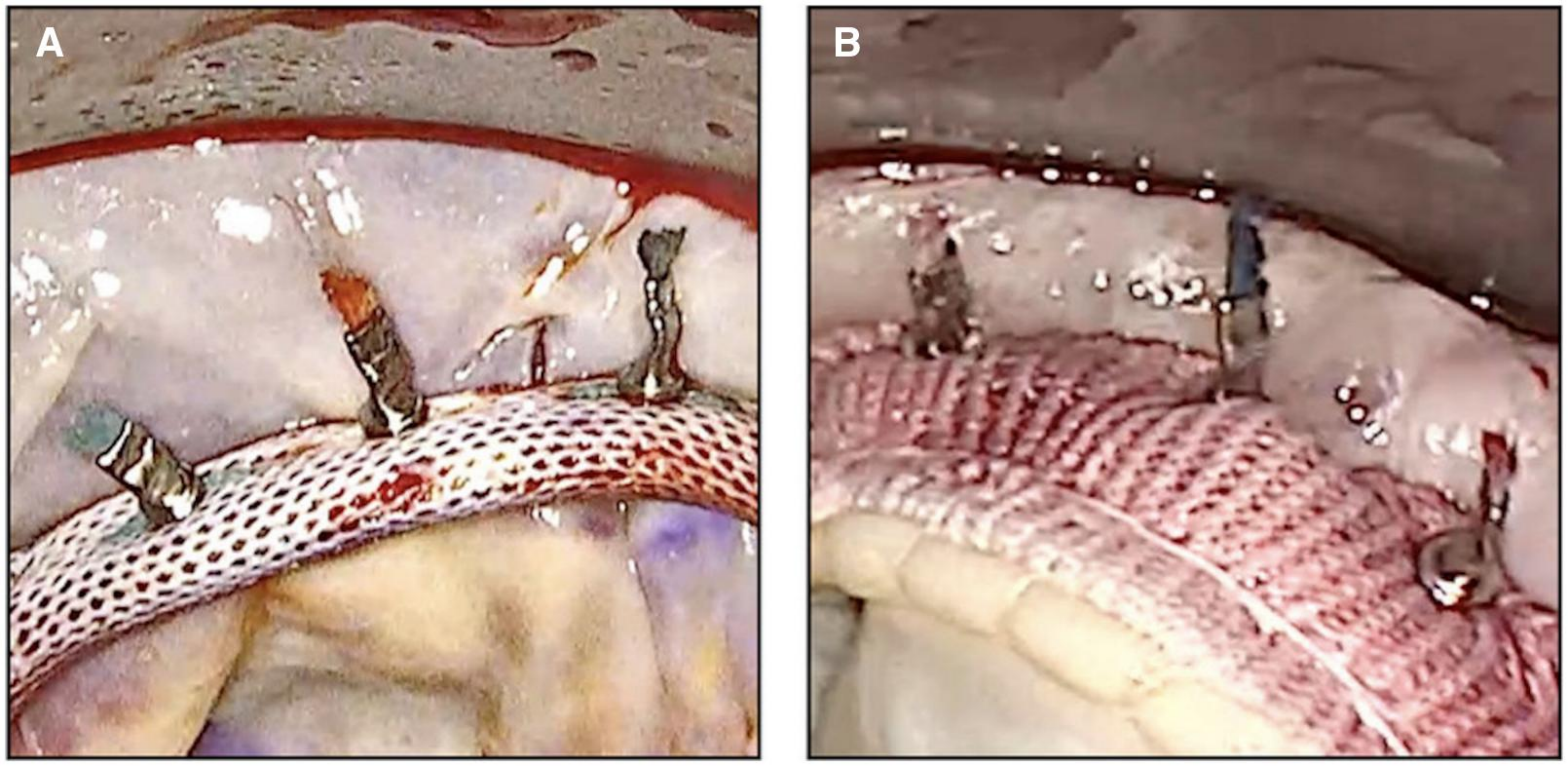
Automated titanium Fasteners Securing Suture in (A) a Mitral Annuloplasty Ring and (B) a Biological Mitral Valve Prosthesis.

In mitral valve surgery, the annuloplasty ring and valve replacement prosthesis are traditionally anchored using hand-tied sutures. However, the tension of these sutures is dependent on the surgeon’s manual technique, introducing variability in suture-holding strength and annular compression. Such inconsistency may lead to uneven mechanical stress distribution along the sewing ring-annulus interface, facilitating the development or progressive enlargement of micro-paravalvular leaks under persistent elevated flow pressure, and ultimately increasing the risk of prosthetic dehiscence over time. The automated suture-securing technology (COR-KNOT, LSI Solutions) is designed to standardize the suture fixation with each fastener deployment, potentially improving the mechanical stability of prosthetic valve implants^1^ (Figure 1). Accordingly, this study investigates the impact of suture-securing techniques on long-term clinical outcomes after mitral valve surgery, with a specific focus on prosthetic dehiscence.

## Patients and Methods

### Ethical statement

The study was conducted following the principles of the Declaration of Helsinki and approved by the Ethics Committee of the Medical University of Vienna (vote number 1158/2024; approval date: April 30, 2024). The requirement for informed consent was waived.

### Study design

This retrospective, single-center study included adult patients who underwent mitral valve repair (MVr) or replacement between November 2008 and November 2024 at the Department of Cardiac and Thoracic Aortic Surgery, Medical University of Vienna. Of the 3440 consecutive patients initially screened, exclusions were made for active or resolved endocarditis, previous or concurrent durable mechanical circulatory support or heart transplantation, surgery for acute type A aortic dissection, ventricular septal defect, or ventricular aneurysm, any congenital heart defect, MVr without annuloplasty ring implantation, incomplete operative data, use of non-CE-certified mitral annuloplasty rings for research purposes or implantation of transcatheter prostheses in mitral position under cardiopulmonary bypass (CPB) (Figure S1). A total of 46 patients (1.3%) had incomplete operative data and were excluded, as per predefined exclusion criteria. Upon applying these criteria, 2678 patients were eligible for analysis and subsequently grouped by the suture-securing technique used: automated titanium fastener group versus hand-tied knots group. The choice of suture-securing technique was dependent on the surgeon’s discretion and was identified through operative reports and institutional protocols. Standardized surgical techniques were employed, using interrupted 2-0 mattress sutures for MVr and pledgeted 2-0 mattress sutures for valve replacement, all anchored to the annulus fibrosus. In all procedures, braided polyester sutures were used (PremiCron; B. Braun Surgical, or Ti-Cron; Covidien). The hand-tied knots group included sutures that were directly tied by hand and those tied indirectly using a knot pusher. The automated titanium fastener was introduced in 2014 at our institution. The device features a slim, ergonomic design and is available in 2 shaft lengths (17 cm and 31 cm), making it suitable for both minimally invasive and open surgical approaches (Figure S2).

### Study end-points and definitions

The primary study end-point was prosthetic dehiscence—defined as dehiscence of either a mitral annuloplasty ring or a mitral valve prosthesis—requiring reintervention. Prosthetic dehiscence refers to the detachment of a valve implant, typically presenting with paravalvular leak, haemolysis, or heart failure. It is considered early when occurring within 30 days of the index procedure.^2^ Endocarditis-related prosthetic dehiscence (active or resolved) was not included in the analysis of the primary outcome. Secondary end-points included ischaemic stroke, intracranial bleeding, and all-cause mortality during the follow-up period. Ischaemic stroke was defined as a neurologic impairment resulting from cerebral infarction due to vascular occlusion, whereas intracranial bleeding denoted radiologically confirmed haemorrhage within the cranial cavity. Mortality data were acquired from the Austrian Federal Statistical Agency. Data collection was obtained through a review of the departmental database and telephone interviews. Patients were censored at 10 years, at the occurrence of each study end-point, or September 28, 2025 (the end of follow-up), whichever came first. Patients who underwent heart transplantation or durable mechanical circulatory support during the follow-up period were censored at the date of the respective operation. A total of 298 patients (11%) were classified as lost to follow-up, with their follow-up time terminated at the date of their last clinical visit. This was primarily attributable to unavailability for patient contact, patients declining to provide further information, or transition of postoperative care to healthcare facilities not part of the regional electronic record system. Study reporting follows the STROBE statement.^3^

### Statistical analysis

Categorical variables were expressed as absolute frequencies and percentages and analyzed using the chi-square test. Continuous variables were tested for normality of distribution and were reported as medians with interquartile ranges (IQR). The comparisons were made using the Mann–Whitney *U*-test. Continuous covariables in the multivariable regression analyses were modelled using functional forms that best represented their distribution. For non-normally distributed covariables, data transformations were applied based on observed distribution patterns to more closely approximate normality.

Univariable and multivariable Fine-Gray proportional subdistribution hazards regression models, accounting for death as a competing event, were used to quantify the risk of prosthetic dehiscence concerning the suture-securing techniques. Covariable selection was guided by a causal inference approach using directed acyclic graphs, integrating both evidence from the published literature and clinical expertise (Figure S3). The following covariables were included in the model: functional mitral valve disease, previous mitral valve surgery, minimally invasive surgery, extensive annular calcification, high-volume mitral valve surgeon (≥25 mitral valve operations annually), and MVr with annuloplasty ring. Cumulative-event curves were generated to depict the time-to-event distribution for prosthetic dehiscence between the study groups. The Firth correction was applied to reduce potential small-sample bias in the parameter estimates due to the limited number of prosthetic dehiscence events. As sensitivity analyses, we conducted inverse probability-weighted regression adjustment, a multivariable competing risk regression with follow-up censored at one year, and a restriction analysis. The effect estimates were consistent with those obtained from the main model.

For the secondary endpoints—ischaemic stroke and intracranial bleeding—multivariable Fine and Gray competing risk regression analysis was performed. The models were adjusted for the covariables: age (years^2^), atrial fibrillation, history of stroke, cerebrovascular disease, minimally invasive surgery, MVr with annuloplasty ring, and cross-clamp time.

In addition, a multivariable Cox proportional hazards regression model was used to assess the effect of the suture-securing technique on all-cause mortality. The model incorporated the covariables: age (years^2^), year of surgery, European System for Cardiac Operative Risk Evaluation II (EuroSCORE II) (log-transformed), previous mitral valve surgery, functional mitral valve disease, minimally invasive surgery, MVr with annuloplasty ring, and cross-clamp time.

Statistical analyses were conducted using SPSS 29 (IBM Corp) and STATA18 (StataCorp). Statistical significance was defined as *P* < .05.

## Results

### Baseline characteristics

Of the included patients, 1072 (40%) underwent mitral valve surgery using an automated titanium fastener device, and 1606 (60%) with conventional hand-tied sutures; Table 1. Patients in the automated titanium fastener group were older (median 69.6, IQR 60.1-76.3 vs median 67.2, IQR 57.7-74.5, *P* < .001). All other baseline characteristics were comparable between the study groups. Echocardiographic features and mitral valve pathology, categorized according to the Carpentier classification, are provided in Table S1.

**Table 1.** Baseline Characteristics

	Automated titanium fastener *N* = 1072 (40%)	Hand-tied knots *N* = 1606 (60%)	*P* value
Age (years), 25th-75th interval	69.6 (60.1-76.3)	67.2 (57.7-74.5)	**<.001**
Gender male, *n* (%)	577 (53.8)	907 (56.5)	.176
EuroSCORE II, 25th-75th interval	3.9 (1.9-8.3)	3.5 (1.6-7.8)	.067
Hypertension, *n* (%)	815 (76.0)	1200 (74.7)	.443
NYHA IV, *n* (%)	96 (9.0)	165 (10.3)	.260
Atrial fibrillation, *n* (%)	534 (49.8)	741 (46.1)	.062
Persistent, *n* (%)	258 (24.1)	379 (23.6)	.780
History of stroke, *n* (%)	67 (6.3)	94 (5.9)	.672
Cerebrovascular disease, *n* (%)	79 (7.4)	121 (7.5)	.874
Diabetes mellitus, *n* (%)	223 (20.8)	290 (18.1)	.077
Dialysis, *n* (%)	25 (2.3)	31 (1.9)	.476
Previous cardiac surgery, *n* (%)	139 (13.0)	184 (11.5)	.240
Previous mitral valve surgery, *n* (%)	88 (8.2)	114 (7.1)	.286
Etiology of native mitral valve disease, *n* (%)			
Isolated degenerative	561 (52.3)	823 (51.2)	.581
Isolated functional	245 (22.9)	383 (23.8)	.552
Isolated rheumatic	69 (6.4)	107 (6.7)	.817
Mixed disease	103 (9.6)	174 (10.8)	.307
Tumor	6 (0.6)	5 (0.3)	.325

Bold indicates statistical significance (*P* < .05).

Abbreviations: EuroSCORE II, European System for Cardiac Operative Risk Evaluation II; NYHA, New York Heart Association Functional Classification.

### Operative characteristics

Minimally invasive surgery was employed less frequently in the automated titanium fastener group (15.7% vs 22.4%, *P* < .001); Table 2. This group had significantly lower rates of MVr with annuloplasty ring implantation (61.4% vs 69.7%, *P* < .001), as well as adjunct techniques including neochordae implantation (19.5% vs 27.8%, *P* < .001), quadrangular resection (3.5% vs 5.7%, *P* = .008), secondary chordae transfer (1.4% vs 2.7%, *P* = .026), and papillary muscle surgery (0.4% vs 1.9%, *P* < .001). Mitral valve replacement was performed more frequently in the automated titanium fastener group (38.6% vs 30.3%, *P* < .001), and preservation of the subvalvular apparatus during this procedure was also more common in this group (30.6% vs 23.4%, *P* < .001). The rate of annular patch reconstruction was significantly higher in the automated titanium fastener group (2.6% vs 1.1%, *P* = .002). Operative times—including CPB time (median 166, IQR 130-211 vs median 176, IQR 139-218, *P* < .001), and cross-clamp time (median 113, IQR 85-141 vs median 118, IQR 92-144, *P* < .001)—were shorter in this group. Furthermore, concomitant procedures were performed more commonly in the automated titanium fastener group, involving aortic valve replacement (21.6% vs 17.7%, *P* = .011), tricuspid valve repair (36.0% vs 29.0%, *P* < .001), left atrial appendage exclusion (28.5% vs 16.3%, *P* < .001), and the use of the epicardial AtriClip device (4.1% vs 0.2%, *P* < .001) (Table S2). Details regarding mitral valve implants, including annuloplasty rings and valve replacement prostheses, are summarized in Table S3.

**Table 2.** Operative Data

	Automated titanium fastener *N* = 1072 (40%)	Hand-tied knots *N* = 1606 (60%)	*P*-value
Indication for surgery-elective, *n* (%)	887 (82.7)	1292 (80.4)	.135
Minimally invasive surgery, *n* (%)	168 (15.7)	359 (22.4)	**<.001**
MVr with annuloplasty ring, *n* (%)^a^	658 (61.4)	1120 (69.7)	**<.001**
Isolated annuloplasty	282 (26.3)	400 (24.9)	.415
Neochordae implantation	209 (19.5)	447 (27.8)	**<.001**
Triangular resection	84 (7.8)	127 (7.9)	.946
Quadrangular resection	37 (3.5)	91 (5.7)	**.008**
Cleft closure	98 (9.1)	154 (9.6)	.698
Commissuroplasty	44 (4.1)	49 (3.1)	.145
Secondary chordae transfer	15 (1.4)	43 (2.7)	**.026**
Papillary muscle surgery	4 (0.4)	30 (1.9)	**<.001**
Alfieri stitch	2 (0.2)	4 (0.2)	.738
Leaflet patch reconstruction	2 (0.2)	1 (0.1)	.346
Commissurotomy	1 (0.1)	4 (0.2)	.360
Mitral valve replacement, *n* (%)	414 (38.6)	486 (30.3)	**<.001**
Mechanical	132 (12.3)	202 (12.6)	.839
Preservation of subvalvular apparatus	328 (30.6)	375 (23.4)	**<.001**
ePTFE artificial sutures for subvalvular	53 (4.9)	85 (5.3)	.689
Continuity			
Annular decalcification, *n* (%)	51 (4.8)	67 (4.2)	.469
Annular patch reconstruction, *n* (%)	28 (2.6)	17 (1.1)	**.002**
Tumor excision, *n* (%)	6 (0.6)	5 (0.3)	.325
High-volume mitral valve surgeon, *n* (%)^b^	531 (49.5%)	838 (52.2%)	.180
Operative times (min), 25th-75th interval			
Cardiopulmonary bypass time	166 (130-211)	176 (139-218)	**<.001**
Cross-clamp time	113 (85-141)	118 (92-144)	**<.001**
Intraoperative MVr failure, *n* (%)	39 (3.6)	55 (3.4)	.769

Bold indicates statistical significance (*P* < .05). Abbreviation: MVr, mitral valve repair.

a When more than one surgical technique was employed, each was documented separately as a distinct variable.

b Surgeon ≥25 mitral valve operations annually.

### Postoperative short-term outcomes and anticoagulant therapy

No significant differences were observed between the study groups with respect to postoperative short-term outcomes and anticoagulant therapy at hospital discharge (Table S4).

### Study end-points

**Figure 2.**
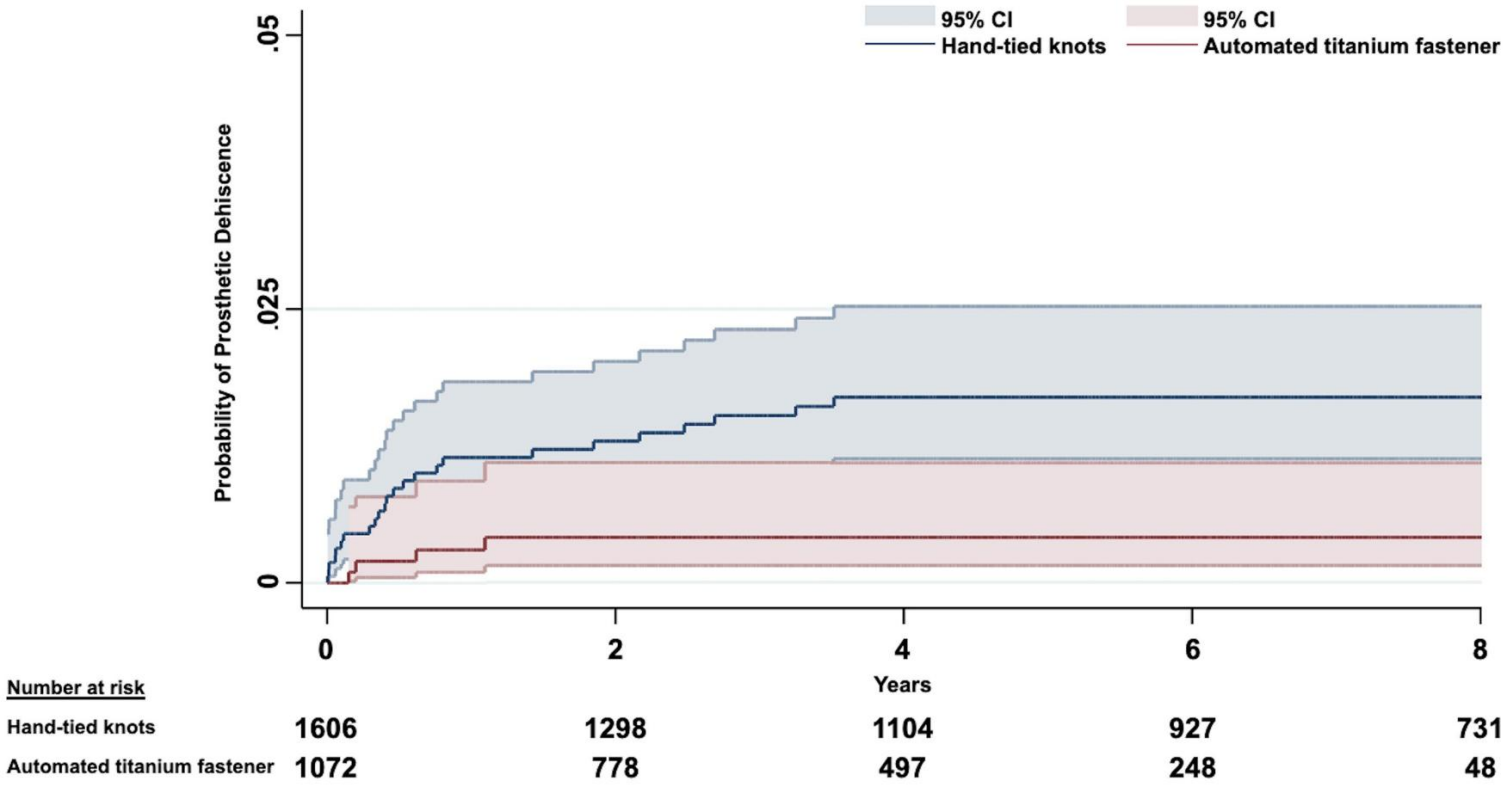
Cumulative Event Curves Demonstrating the Probability of Prosthetic Dehiscence Between the Automated Titanium Fastener Group and the Hand-Tied Knots Group. Abbreviation: CI, confidence interval.

A total of 31 patients (1.2%) had prosthetic dehiscence during the follow-up period. The median follow-up duration was 5.3 years (IQR 2.3-8.7). The use of an automated titanium fastener device was associated with a significantly lower risk of prosthetic dehiscence during follow-up in both the univariable (crude sub-hazard ratio [sHR] 0.32; 95% confidence interval [CI] 0.12-0.86, *P* = .023) and multivariable competing risk regression analysis (adjusted sHR 0.34; 95% CI, 0.12-0.91, *P* = .033) (Table 3). Cumulative incidence curves for both study groups are depicted in Figure 2. In the Firth-corrected multivariable cause-specific regression model, the main effect showed an independent protective association after penalized adjustment (HR 0.37; 95% CI, 0.13-0.87, *P* = .021).

**Table 3.** Primary Study End-Point—Prosthetic Dehiscence.^a^

	sHR	95% CI	*P* value
**Univariable relative effects**			
Automated titanium fastener	0.32	0.12-0.86	**.023**
**Multivariable relative effects**			
Automated titanium fastener	0.34	0.12-0.91	**.033**
Functional mitral valve disease	1.58	0.77-3.26	.216
Previous mitral valve surgery	2.70	0.76-9.57	.125
Minimally invasive surgery	0.81	0.31-2.10	.664
Extensive annular calcification	2.23	0.76-6.50	.143
High-volume mitral valve surgeon	0.79	0.39-1.61	.511
Mitral valve repair with annuloplasty ring	2.25	0.81-6.29	.121

Bold indicates statistical significance (*P* < .05). Abbreviations: CI, confidence interval; sHR, subhazard ratio.

a Effects calculated as sHR based on a competing risk regression analysis.

Throughout the follow-up period, 163 patients (6.1%) had an ischaemic stroke, while 69 patients (2.6%) were diagnosed with intracranial bleeding. The median follow-up duration was 5.1 years (IQR 2.1-8.6) for ischaemic stroke and 5.3 years (IQR 2.4-8.8) for intracranial bleeding. The use of an automated titanium fastener was not associated with an increased risk of ischaemic stroke (adjusted sHR 0.92; 95% CI, 0.67-1.27, *P* = .600) as well as intracranial bleeding (adjusted sHR 0.89; 95% CI, 0.52-1.52, *P* = .675) during follow-up, as demonstrated in the multivariable proportional competing risk regression analysis (Tables S5 and S6).

Overall, 718 patients (26.8%) died during the follow-up period. The median follow-up duration for all-cause mortality was 5.3 years (IQR 2.4-8.9). Based on multivariable Cox proportional hazards regression analysis, the use of the automated titanium fastener device was not associated with an increased risk of all-cause mortality (adjusted HR 0.93; 95% CI, 0.74-1.18, *P* = .559) (Table S7).

### Characteristics of prosthetic dehiscence

The overall rate of prosthetic dehiscence—including both mitral annuloplasty ring and valve replacement prosthesis—was significantly lower in the automated titanium fastener group (0.5% vs 1.6%, *P* = .006), as was the rate of annuloplasty ring dehiscence specifically (0.5% vs 1.9%, *P* = .012) (Table 4). Annuloplasty ring dehiscence with a semi-rigid ring was observed less frequently in the automated fastener group (0.5% vs 1.5%, *P* = .040). In terms of temporal classification, late-onset prosthetic dehiscence was also less common in this group (0.5% vs 1.3%, *P* = .030). In the automated titanium fastener group, 2 patients were successfully treated with percutaneous paravalvular leak closure during the follow-up period. In contrast, all patients in the hand-tied knot group underwent surgical reintervention. In the knot-pusher subgroup, 3 patients had prosthetic dehiscence requiring reintervention. During the follow-up period, prosthetic dehiscence was not detected in patients who underwent annular patch reconstruction at the index procedure (restricted analysis: adjusted sHR 0.34; 95% CI, 0.13-0.93, *P* = .035). Among patients with annular decalcification, 3 prosthetic dehiscence events were observed, with the effect estimate for automated titanium fastener: adjusted sHR 0.32 (95% CI, 0.12-0.87, *P* = .026).

**Table 4.** Characteristics of Prosthetic Dehiscence

	Automated titanium fastener *N* = 1072 (40 %)	Hand-tied knots *N* = 1606 (60 %)	*P* value
**Prosthetic dehiscence, *n* (%)**	5 (0.5)	26 (1.6)	**.006**
Annuloplasty ring dehiscence	3 (0.5)	21 (1.9)	**.012**
Flexible	0 (0)	0 (0)	–
Semi-rigid	3 (0.5)	17 (1.5)	**.040**
Rigid	0 (0)	4 (0.4)	.125
Valve replacement prosthesis	2 (0.5)	5 (1.0)	.353
dehiscence			
**Onset, *n* (%)**			
Early-onset	0 (0)	5 (0.3)	.067
Late-onset	5 (0.5)	21 (1.3)	**.030**
**Anatomical site, *n* (%)**			
Anterior annulus	2 (0.2)	6 (0.4)	.385
Posterior annulus	2 (0.2)	11 (0.7)	.069
Anterior and posterior	1 (0.1)	6 (0.4)	.164
Commissural	0 (0)	3 (0.2)	.157

Bold indicates statistical significance (*P* < .05).

### Primary causes of MVr and replacement failure requiring reintervention

The rate of artificial chordae dehiscence was lower in the automated titanium fastener group (0.0% vs 0.9%, *P* = .015) (Table S8).

### Non-mitral valve-related reinterventions

Non-mitral valve-related reinterventions had comparable rates between the study groups (Table S9).

## Discussion

This study represents the first large-scale, comparative analysis assessing the long-term outcomes with respect to the suture-securing techniques used in mitral valve surgery. The results indicate that the automated suture-securing technology is associated with a significantly lower risk of prosthetic dehiscence requiring reintervention during follow-up. This observed reduction may be attributed to the mechanical consistency and uniformity of suture tension along the prosthetic sewing ring, achieved with the automated titanium fastener device. In contrast, hand-tied knots, though widely used and reliable, are inherently prone to operator-dependent variability in suture-holding tension and may vary based on mitral valve exposure and angulation in the same surgeon.^1^

In an experimental study, Lee et al. concluded that sutures secured with automated titanium fasteners exhibited significantly greater suture-holding pressure than manually tied knots (*P* < .001). Moreover, suture consistency, stated as attachment pressure variability, was significantly lower in the automated titanium fastener group; *P* = .040.^1^ Complementing these findings, a digital pressure mapping study of surgically implanted aortic valve prostheses systematically evaluated the attachment strength of automated titanium fasteners versus hand-tied knots, identifying pressure values below 80 mmHg as indicative of an increased risk of paravalvular leak. Both intrasuture and extrasuture pressures falling below this threshold were significantly more frequent in hand-tied groups (*P* < .001 for both), suggesting that automated titanium fasteners may improve mechanical stability by ensuring more secure prosthetic fixation.^4^ An *in vivo* ovine study comparing histological outcomes between suture-securing techniques showed no significant differences in inflammatory response or pathological lesions. Importantly, no cases of tissue necrosis or tearing were documented in the automated fastener group.^5^

Indeed, prosthetic dehiscence may originate from subtle mechanical instability caused by inconsistent and insufficient suture tension along the sewing ring. Under the repetitive haemodynamic loading of the cardiac cycle, as well as twisting contraction of the left ventricle in combination with tilting mechanics of the annulus, localized areas of uneven suture tension are exposed to elevated shear forces, which may initiate micro-separation between the native annulus and mitral implant (annuloplasty ring or valve replacement prosthesis).^6^^,^^7^ As regurgitant jet passes through the developing micro-separations, turbulent wall-shear stress may further weaken tissue integrity, thereby creating a self-reinforcing mechanical and haemodynamic feedback loop that may ultimately result in overt dehiscence.^8^ In this study, the rate of late-onset prosthetic dehiscence was significantly lower in the automated titanium fastener group (0.5% vs 1.3%, *P* = .030). We believe that the strengthened mechanical stability achieved by the automated titanium fastener device may serve as a strategy to improve long-term durability of annuloplasty rings and valve prostheses in mitral valve surgery.

Interestingly, the mechanical instability-driven progression reflects a broader physical principle observed in natural hydrodynamic systems, where minor instability of channel structure under continuous high flow pressure can initiate the formation of a side channel. As flow diverts into the emerging path, localized shear stress increases, further eroding the weakest area and amplifying the initial disturbance over time.^9^

During surgical implantation, incomplete or inadequately tensioned knots, often referred to as “air knots,” may inadvertently occur with hand-tying.^10^ Though they may appear intact upon inspection, such knots fail to ensure optimal mechanical tension or tissue compression at the sewing ring–annulus interface and may serve as niduses for dehiscence under sustained dynamic flow stress. In contrast, the automated titanium fastener device is designed to standardize the suture-fixation quality and eliminate tension variability with each deployment.^1^

Limited exposure, such as in minimally invasive or redo procedures, can impair visualization and reduce maneuverability, increasing the technical difficulty of precise knot tying. In this context, automated titanium fasteners facilitate secure fixation from remote or restricted access angles.^10^^,^^11^

Concerning the anatomical location of prosthetic dehiscence, Pierce et al. demonstrated in an experimental ovine model that local shear forces on sutures increase during systole and vary by annular region, with higher forces identified in the anterior segment. Furthermore, suture holding strength was greater in this segment, likely due to higher collagen fibre density.^12^ In our study, no significant differences in the anatomical site of prosthetic dehiscence were found between the study groups.

When specifically addressing mitral annuloplasty ring dehiscence, previous studies reported that semi-rigid rings with a saddle-shaped design provide superior conformity to physiological annular geometry with more favourable mechanical stress distribution, potentially reducing the risk of dehiscence.^13^ Within the analyzed cohort, the rate of dehiscence involving semi-rigid rings was significantly lower in the automated fastener group (0.5% vs 1.5%, *P* = .040), indicating that mechanical vulnerability and susceptibility to dehiscence may be affected by the suture-securing technique. Gillinov et al. noted that annuloplasty ring dehiscence is inherently unpredictable and may occur regardless of the MVr technique or type of implanted annuloplasty ring.^14^

Importantly, even small subclinical paravalvular leaks may alter intracardiac flow dynamics and may lead to substantial haemodynamic deterioration.^15^ The regurgitant jets generate high-velocity, eccentric flow patterns that disrupt physiological vortex formation within the left ventricle, resulting in increased energy dissipation. These conditions can contribute to progressive cardiac remodelling, diastolic dysfunction, and ultimately, congestive heart failure.^16–18^

Previous research has demonstrated that the use of an automated titanium fastener device is not associated with an increased risk of stroke, intracranial bleeding, or mortality,^19^^,^^20^ which is in line with the present findings.

Nevertheless, the integration of automated suture-securing technology into routine surgical practice is influenced by cost-effectiveness, which remains an important aspect for healthcare institutions.

## Limitations

This study is a retrospective, single-centre analysis. As such, the results may not be fully generalizable to other centres. Although multivariable regression analysis can alleviate confounding to some extent, residual and unmeasured confounding cannot be fully ruled out. Intraoperative transoesophageal echocardiography findings, such as minor paravalvular leak or mild residual transvalvular leak, were not assessed. Detailed information on suture placement technique (everting vs non-everting) for mitral valve replacement was not available. The absence of standardized echocardiographic follow-up examination in all patients restricts the ability to identify subclinical or minor paravalvular leaks. Prosthetic dehiscence may occur due to the fragility of the tissue or technical aspects of the procedure itself, such as superficial suture placement.

## Conclusion

The use of an automated titanium fastener device seems to be associated with a significantly lower risk of prosthetic dehiscence in mitral valve surgery. Given the limited number of prosthetic dehiscence events and the inherent susceptibility of observational analyses to residual confounding, the results of this study should be interpreted with caution and considered hypothesis-generating. A randomized controlled trial is warranted to confirm these findings.

## Supplementary Material

ezag013_Supplementary_Data

## Data Availability

Anonymized datasets will be shared on reasonable request to the corresponding author.
