# Supplementary material for: Automated Suture Securing Technology in Mitral Valve Surgery: A Strategy to Reduce Prosthetic Dehiscence?
Source: Eur J Cardiothorac Surg. 2026 Jan 9;68(1):ezag013. doi: 10.1093/ejcts/ezag013 (PMC12816918; doi:10.1093/ejcts/ezag013)
Supplement: ezag013_Supplementary_Data [file ezag013_supplementary_data.doc]

**Supplemental Figure S1.** Study flowchart

**
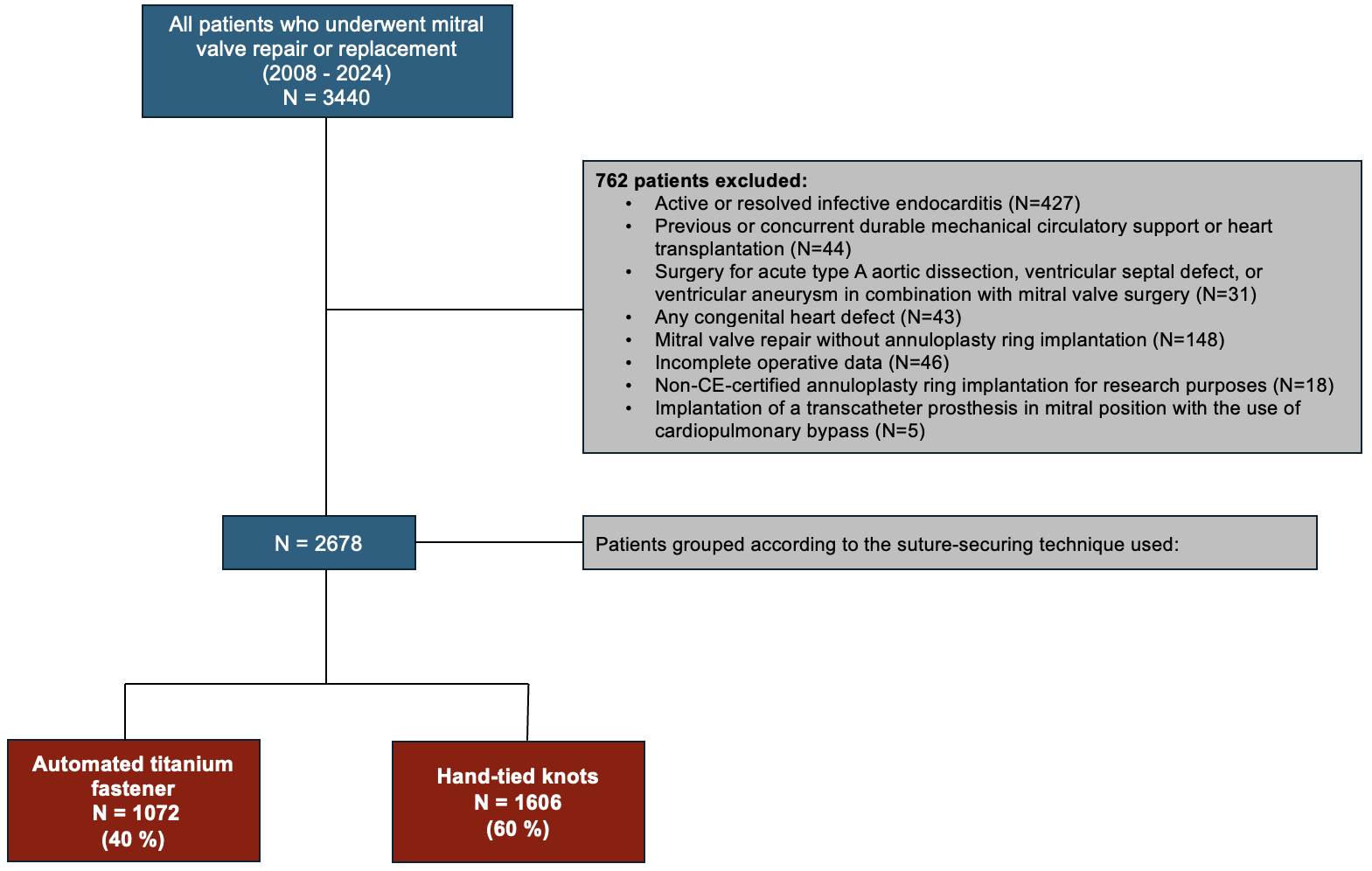
**

**Supplemental Figure S2.** The automated titanium fastener device features a rotational knob with an indicator fin, a white handle, and a purple lever located at the proximal end (A). At the distal end of the shaft, the loading unit (B) is positioned, allowing the automated titanium fastener to be maneuvered within the shaft. The distal tip of the device is advanced over the partially tensioned suture to the target site. The suture slot and indicator fin are aligned with the center of the prosthesis, and activation of the purple lever deploys the titanium fastener, simultaneously securing and trimming the suture in a single step. Figure reproduced with permission from LSI Solutions (https://www.lsisolutions.com/products/1/cor-knot-device/).

**
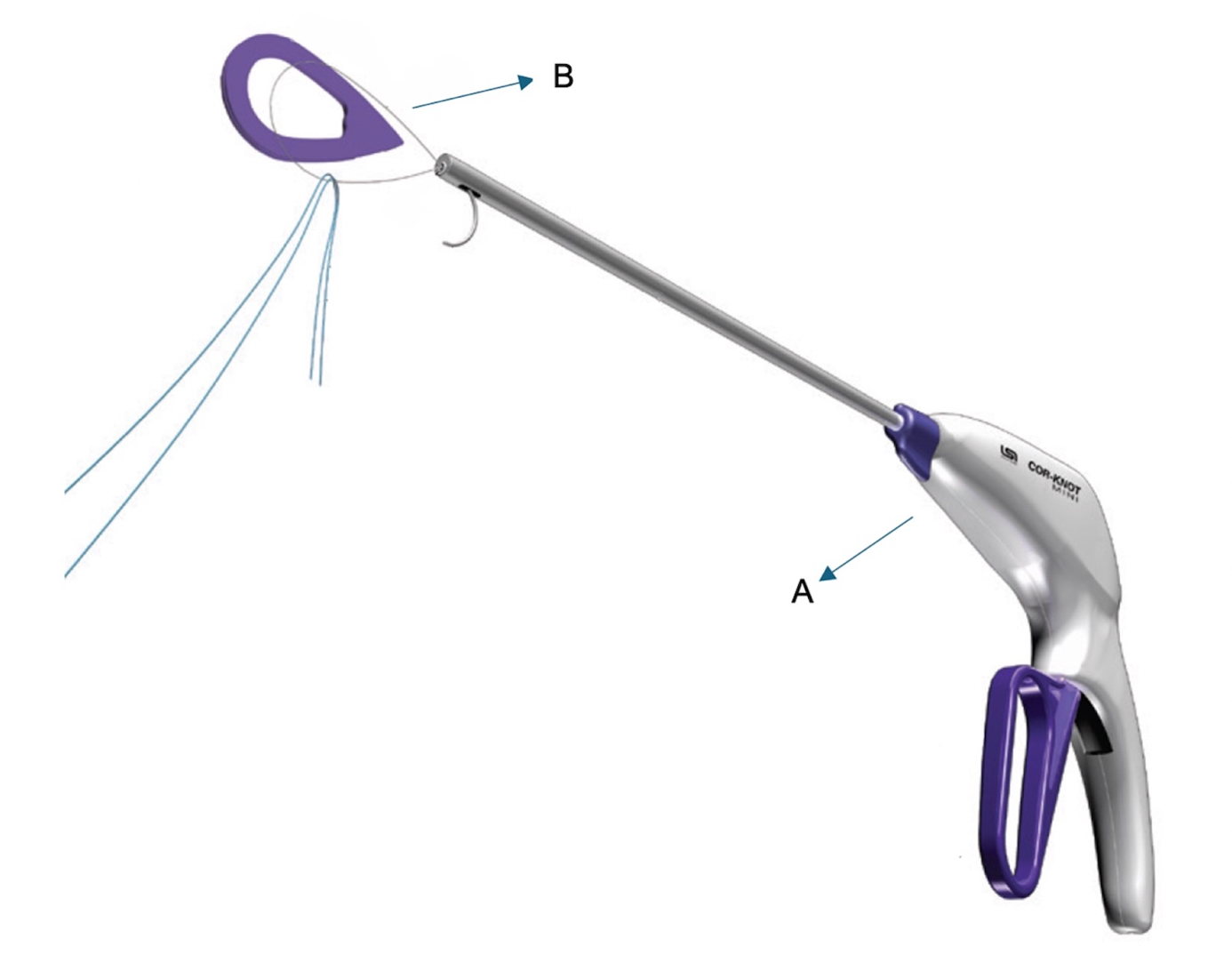
**

**Supplemental Figure S3.** Directed acyclic graph illustrating covariable adjustment sets for the analysis of the primary endpoint.

**
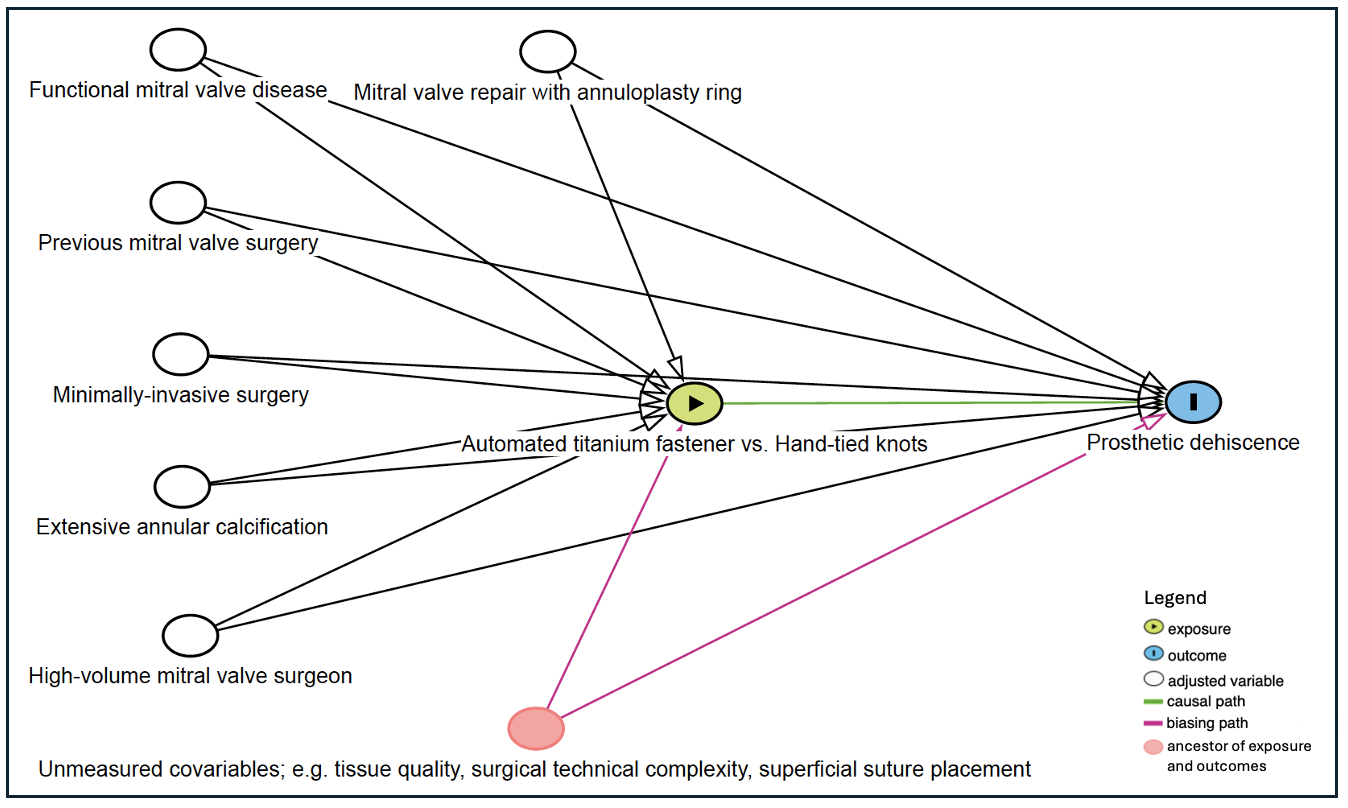
**

**Supplemental Table S1. Echocardiographic characteristics and Carpentier classification of mitral valve disease**

|  | **Automated titanium fastener**  **N = 1072**  **(40 %)** | | | **Hand-tied**  **knots**  **N = 1606**  **(60 %)** | **p-value** |
| --- | --- | --- | --- | --- | --- |
| Ejection fraction, n (%) |  | | |  |  |
| Good >50% | 791 (73.8) | | | 1132 (70.5) | 0.063 |
| Moderate 31–50% | 225 (21.0) | | | 379 (23.6) | 0.113 |
| Poor 21–30% | 43 (4.0) | | | 68 (4.2) | 0.777 |
| Very poor <20% | 13 (1.2) | | | 27 (1.7) | 0.327 |
| Pulmonary hypertension, n (%) | | | | |  |
| Moderate (SPAP 31-55 mmHg) | | 421 (39.3) | | 624 (38.9) | 0.828 |
| Severe (SPAP >55 mmHg) | 288 (26.9) | | | 539 (33.6) | **<0.001** |
| **Carpentier classification*** | | | | | |
| *Type I,* n (%) | 354 (33.0) | | | 541 (33.7) | 0.721 |
| Anular dilatation | 248 (23.1) | | | 407 (25.3) | 0.193 |
| Perforation | 11 (1.0) | | | 14 (0.9) | 0.684 |
| Cleft | 120 (11.2) | | | 175 (10.9) | 0.810 |
| *Type II*, n (%) | 498 (46.5) | | | 837 (52.1) | **0.004** |
| Anterior leaflet prolapse | 164 (15.3) | | | 283 (17.6) | 0.114 |
| Posterior leaflet prolapse | 399 (37.2) | | | 689 (42.9) | **0.003** |
| Commissural prolapse | 26 (2.4) | | | 70 (4.4) | **0.008** |
| *Type IIIa*, n (%) | 257 (24.0) | | | 345 (21.5) | 0.130 |
| Rheumatic | 89 (8.3) | | | 136 (8.5) | 0.879 |
| Calcified disease | 177 (16.5) | | | 238 (14.8) | 0.236 |
| Extensive annular calcification | | | 116 (10.8) | 148 (9.2) | 0.172 |
| Leaflet calcification | | 126 (11.8) | | 174 (10.8) | 0.460 |
| Subvalvular calcification | 12 (1.1) | | | 32 (2.0) | 0.082 |
| *Type IIIb*, n (%) | 108 (10.1) | | | 151 (9.4) | 0.564 |
| Tumor, n (%) | 6 (0.6) | | | 5 (0.3) | 0.325 |
| Prior mitral valve surgery or intervention, n (%) | | | |  |  |
| Prior mitral valve replacement | 17 (1.6) | | | 24 (1.5) | 0.850 |
| Prior mitral valve repair | 39 (3.6) | | | 66 (4.1) | 0.538 |
| Prior surgical commissurotomy | 0 (0) | | | 2 (0.1) | 0.248 |
| Prior transcatheter MVr | 19 (1.8) | | | 2 (0.1) | **<0.001** |
| Prior balloon valvuloplasty | 13 (1.2) | | | 20 (1.2) | 0.940 |

Bold indicates statistical significance (p < 0.05).

* In cases of mitral valve disease involving multiple pathologies (e.g., annular dilation and leaflet prolapse), each pathology was documented separately and considered individually in the analysis.

MVr: Mitral valve repair, SPAP: Systolic pulmonary artery pressure.

**Supplemental Table S2.** **Concomitant procedures**

|  | **Automated titanium fastener**  **N = 1072**  **(40 %)** | | **Hand-tied**  **knots**  **N = 1606**  **(60 %)** | **p-value** |
| --- | --- | --- | --- | --- |
| Aortic valve replacement, n (%) | 232 (21.6) | | 284 (17.7) | **0.011** |
| Aortic valve repair, n (%) | 11 (1.0) | | 10 (0.6) | 0.246 |
| Tricuspid valve replacement, n (%) | 11 (1.0) | | 7 (0.4) | 0.067 |
| Tricuspid valve repair, n (%) | 386 (36.0) | | 466 (29.0) | **<0.001** |
| Pulmonary valve replacement, n (%) | 2 (0.2) | | 0 (0) | 0.083 |
| Aortic root surgery*, n (%) | 17(1.6) | | 17 (1.1) | 0.232 |
| Aortic root enlargement, n (%) | 12 (1.1) | | 17 (1.1) | 0.881 |
| Reduction aortoplasty, n (%) | 18 (1.7) | | 28 (1.7) | 0.900 |
| Supracoronary ascending aortic replacement, n (%) | 7 (0.7) | | 15 (0.9) | 0.430 |
| CABG, n (%) | 258 (24.1) | | 403 (25.1) | 0.546 |
| Atrial fibrillation surgery, n (%) | 234 (21.8) | | 362 (22.5) | 0.664 |
| Left atrial appendage exclusion, n (%) | | 306 (28.5) | 262 (16.3) | **<0.001** |
| Epicardial AtriClip device, n (%) | 44 (4.1) | | 3 (0.2) | **<0.001** |
| Atrial septal defect closure, n (%) | 79 (7.4) | | 139 (8.7) | 0.233 |

Bold indicates statistical significance (p < 0.05).

* Aortic root surgery involves any of the following: Bentall procedure, valve-sparing aortic root replacement, or implantation of a homograft.

CABG: Coronary artery bypass grafting.

**Supplemental Table S3. Mitral valve implants (annuloplasty ring and valve replacement prostheses)**

|  | **Automated titanium fastener**  **N = 1072**  **(40 %)** | | | **Hand-tied**  **knots**  **N = 1606**  **(60 %)** | **p-value** | |
| --- | --- | --- | --- | --- | --- | --- |
| **Implanted annuloplasty ring, n (%)** |  | | |  | |  |
| Edwards Physio II Annuloplasty Ring | 631 (58.9) | | | 923 (57.5) | | 0.475 |
| Edwards Physio Annuloplasty Ring | 0 (0) | | | 62 (3.9) | | **<0.001** |
| Edwards Classic Annuloplasty Ring | 0 (0) | | | 5 (0.3) | | 0.067 |
| Edwards IMR ETlogix Annuloplasty Ring | 20 (1.9) | | | 50 (3.1) | | **0.047** |
| Cosgrove-Edwards Annuloplasty System | 0 (0.0) | | | 4 (0.2) | | 0.102 |
| Sorin MEMO 4D™ Ring | 2 (0.2) | | | 12 (0.7) | | **0.049** |
| Medtronic CG Future® Annuloplasty Ring | 1 (0.1) | | | 23 (1.4) | | **<0.001** |
| Medtronic CG Future® Annuloplasty Band | 4 (0.4) | | | 8 (0.5) | | 0.635 |
| enCor™ Mitral Valve Repair System | 0 (0) | | | 30 (1.9) | | **<0.001** |
| Puig-Massana-Shiley Annuloplasty Ring | 0 (0) | | | 2 (0.1) | | 0.248 |
| **Implanted replacement prosthesis, n (%)** |  | | |  | |  |
| Edwards PERIMOUNT Bioprosthesis | 0 (0) | | | 6 (0.4) | | **0.045** |
| Edwards PERIMOUNT Magna Bioprosthesis | 1 (0.1) | | | 32 (2.0) | | **<0.001** |
| Edwards PERIMOUNT Magna Ease Bioprosthesis | | | 37 (3.5) | 45 (2.8) | | 0.339 |
| Edwards MITRIS RESILIA™ Mitral Bioprosthesis | | 9 (0.8) | | 2 (0.1) | | 0.005 |
| Medtronic Mosaic™ Mitral Bioprosthesis | 218 (20.3) | | | 196 (12.2) | | **<0.001** |
| Abbott Epic™ Valve | 17 (1.6) | | | 3 (0.2) | | **<0.001** |
| On-X® Mechanical | 129 (12.0) | | | 173 (10.8) | | 0.312 |
| SJM® Masters Series Mechanical | 3 (0.3) | | | 21 (1.3) | | **0.006** |
| Medtronic ADVANTAGE™ Mechanical | 0 (0) | | | 8 (0.5) | | **0.021** |

Bold indicates statistical significance (p < 0.05).

**Supplemental Table S4. Postoperative short-term outcomes and Anticoagulant Therapy**

|  | **Automated titanium fastener**  **N = 1072**  **(40 %)** | | **Hand-tied**  **knots**  **N = 1606**  **(60 %)** | **p-value** |
| --- | --- | --- | --- | --- |
| **Postoperative short-term outcomes** | | | | |
| Prolonged intensive care unit stay >7 days, n (%) | | 215 (20.1) | 325 (20.2) | 0.909 |
| Atrial fibrillation, n (%) | 492 (45.9) | | 695 (43.3) | 0.181 |
| Dialysis, n (%) | 72 (6.7) | | 92 (5.7) | 0.296 |
| Temporary mechanical circulatory support, n (%) | 71 (6.6) | | 103 (6.4) | 0.829 |
| Re-exploration for bleeding, n (%) | 87 (8.1) | | 107 (6.7) | 0.155 |
| 30-day stroke, n (%) | 33 (3.1) | | 56 (3.5) | 0.563 |
| 30-day new permanent pacemaker implantation, n (%) | | 96 (9.0) | 113 (7.0) | 0.070 |
| 30-day mortality, n (%) | 31 (2.9) | | 58 (3.6) | 0.309 |
| **Anticoagulant Therapy** |  | |  |  |
| Vitamin K antagonists, n (%) | 131 (12.2) | | 183 (11.4) | 0.529 |
| Direct oral anticoagulants, n (%) | 301 (28.1) | | 403 (25.1) | 0.097 |
| Parenteral anticoagulants, n (%) | 592 (55.2) | | 924 (57.5) | 0.258 |

**Supplemental Table S5: Secondary study endpoint – Ischemic stroke ^a^**

| **Variables** | **sHR** | | **95% CI** | **p-value** |
| --- | --- | --- | --- | --- |
| Automated titanium fastener | 0.92 | | 0.67 – 1.27 | 0.600 |
| Age (years^2^) | 1.00 | | 1.00 – 1.00 | 0.911 |
| Atrial fibrillation | 1.04 | | 0.76 – 1.42 | 0.829 |
| History of stroke | 1.40 | | 0.82 – 2.41 | 0.222 |
| Cerebrovascular disease | 1.06 | | 0.62 – 1.83 | 0.822 |
| Minimally invasive surgery | | 0.38 | 0.21 – 1.02 | 0.052 |
| Mitral valve repair with annuloplasty ring | | 0.92 | 0.66 – 1.28 | 0.624 |
| Cross-clamp time | 1.00 | | 1.00 – 1.01 | **0.001** |

Bold indicates statistical significance (p < 0.05).

a) Effects calculated as sHR based on a multivariable proportional competing risk regression model.

CI: confidence interval, sHR: subhazard ratio.

**Supplemental Table S6: Secondary study endpoint – Intracranial bleeding ^a^**

| **Variables** | **sHR** | | **95% CI** | **p-value** |
| --- | --- | --- | --- | --- |
| Automated titanium fastener | 0.89 | | 0.52 – 1.52 | 0.675 |
| Age (years^2^) | 1.00 | | 1.00 – 1.00 | **0.013** |
| Atrial fibrillation | 1.00 | | 0.62 – 1.61 | 0.992 |
| History of stroke | 1.04 | | 0.41 – 2.61 | 0.934 |
| Cerebrovascular disease | 0.90 | | 0.38 – 2.14 | 0.813 |
| Minimally invasive surgery | | 0.40 | 0.15 – 1.04 | 0.061 |
| Mitral valve repair with annuloplasty ring | | 0.77 | 0.48 – 1.25 | 0.295 |
| Cross-clamp time | 1.00 | | 1.00 – 1.01 | 0.092 |

Bold indicates statistical significance (p < 0.05).

a) Effects calculated as sHR based on a multivariable proportional competing risk regression model.

CI: confidence interval, sHR: subhazard ratio.

**Supplemental Table S7: Secondary study endpoint – All-cause mortality ^a^**

| **Variables** | **HR** | | **95% CI** | **p-value** |
| --- | --- | --- | --- | --- |
| Automated titanium fastener | 0.93 | | 0.74 – 1.18 | 0.559 |
| Age (years^2^) | 1.00 | | 1.00 – 1.01 | **<0.001** |
| Year of surgery | 0.97 | | 0.95 – 0.99 | **0.039** |
| EuroSCORE II (log-transformed) | 1.79 | | 1.63 – 1.97 | **<0.001** |
| Previous mitral valve surgery | 0.91 | | 0.73 – 1.13 | 0.394 |
| Functional mitral valve disease | 1.27 | | 1.06 – 1.52 | **0.010** |
| Minimally invasive surgery | 0.42 | | 0.30 – 0.61 | **<0.001** |
| Mitral valve repair with annuloplasty ring | | 0.77 | 0.64 – 0.93 | **0.005** |
| Cross-clamp time | 1.00 | | 1.00 – 1.01 | **<0.001** |

Bold indicates statistical significance (p < 0.05).

a) Effects calculated as HR based on a multivariable Cox proportional hazards regression model.

CI: confidence interval, EuroSCORE II: European System for Cardiac Operative Risk Evaluation II, HR: hazard ratio.

**Supplemental Table S8. Primary causes of mitral valve repair and replacement failure requiring reintervention**

|  | | **Automated titanium fastener**  **N = 1072**  **(40 %)** | | **Hand-tied**  **knots**  **N = 1606**  **(60 %)** | **p-value** | |
| --- | --- | --- | --- | --- | --- | --- |
| MVr with annuloplasty ring, n (%) | 658 (61.4) | | 1120 (69.7) | | | **<0.001** |
| **Mitral valve repair failure, n (%)** | | | | | | |
| Progressive primary valve disease | | 7 (1.1) | | 18 (1.6) | | 0.347 |
| Annuloplasty ring dehiscence | | 3 (0.5) | | 21 (1.9) | | **0.012** |
| Endocarditis | | 4 (0.6) | | 10 (0.9) | | 0.512 |
| Artificial chordae dehiscence | | 0 (0) | | 10 (0.9) | | **0.015** |
| Systolic anterior motion | | 2 (0.3) | | 4 (0.4) | | 0.852 |
| Incomplete repair | | 2 (0.3) | | 2 (0.2) | | 0.590 |
| Fibrosis | | 0 (0) | | 2 (0.2) | | 0.278 |
| Mitral valve replacement, n (%) | | 414 (38.6) | | 486 (30.3) | | **<0.001** |
| **Mitral valve replacement failure, n (%)** | |  | |  | |  |
| Structural valve deterioration | | 3 (0.7) | | 7 (1.4) | | 0.307 |
| Non-structural valve deterioration | | 4 (1.0) | | 1 (0.2) | | 0.126 |
| Valve replacement prosthesis | | 2 (0.5) | | 5 (1.0) | | 0.353 |
| dehiscence | |  | |  | |  |
| Thrombosis | | 3 (0.7) | | 1 (0.2) | | 0.243 |
| Endocarditis | | 5 (1.2) | | 9 (1.9) | | 0.436 |

Bold indicates statistical significance (p < 0.05).

**Supplemental Table S9. Non-mitral valve–related reint**erventions

|  | | **Automated titanium fastener**  **N = 1072**  **(40 %)** | | | | **Hand-tied**  **knots**  **N = 1606**  **(60 %)** | **p-value** |
| --- | --- | --- | --- | --- | --- | --- | --- |
| Aortic valve surgery/intervention, n (%) |  | | | | |  |  |
| Aortic valve surgery | 3 (0.3) | | | | | 12 (0.7) | 0.112 |
| Transcatheter aortic valve implantation | | | 3 (0.3) | | | 8 (0.5) | 0.387 |
| Tricuspid valve surgery/intervention, n (%) | | |  | | |  |  |
| Tricuspid valve surgery | 2 (0.2) | | | | | 3 (0.2) | 0.999 |
| Tricuspid transcatheter edge-to-edge repair | | | | | 3 (0.3) | 3 (0.2) | 0.618 |
| CABG, n (%) | 0 (0) | | | | | 2 (0.1) | 0.248 |
| Durable mechanical circulatory support, n (%) | | | | 4 (0.4) | | 5 (0.3) | 0.787 |
| Heart transplantation, n (%) | 0 (0) | | | | | 5 (0.3) | 0.067 |
| Ventricular septal defect closure, n (%) | 0 (0) | | | | | 1 (0.1) | 0.414 |
| Ascending aortic surgery, n (%) | 2 (0.2) | | | | | 5 (0.3) | 0.536 |
| Ventriculoplasty, n (%) | 0 (0) | | | | | 2 (0.1) | 0.248 |

Bold indicates statistical significance (p < 0.05).

CABG: Coronary artery bypass grafting.
